# Probabilities of False Alarm for Vital Sign Detection on the Basis of a Doppler Radar System

**DOI:** 10.3390/s18030694

**Published:** 2018-02-26

**Authors:** Nguyen Thi Phuoc Van, Liqiong Tang, Subhas Chandra Mukhopadhyay, Duc Minh Nguyen, Faraz Hasan

**Affiliations:** 1 School of Engineering and Advanced Technology, Massey University, Manawatu Private Bag 11 222, Palmerston North 4442, New Zealand; V.Nguyen@massey.ac.nz (N.T.P.V.); L.Tang@massey.ac.nz (L.T.); F.Hasan@massey.ac.nz (F.H.);; 2 School of Engineering, Macquarie University, Sydney 2109, Australia; 3 School of Electronics and Telecommunications, Hanoi University of Science and Technology, Hanoi 100000, Vietnam; minh.nguyenduc1@hust.edu.vn

**Keywords:** vital signals, finding survivors, Doppler radar, heart beat, breathing signal, detection probability

## Abstract

Vital detection on the basis of Doppler radars has drawn a great deal of attention from researchers because of its high potential for applications in biomedicine, surveillance, and finding people alive under debris during natural hazards. In this research, the signal-to-noise ratio (SNR) of the remote vital-sign detection system is investigated. On the basis of different types of noise, such as phase noise, Gaussian noise, leakage noise between the transmitting and receiving antennae, and so on, the SNR of the system has first been examined. Then the research has focused on the investigation of the detection and false alarm probabilities of the system when the transmission link between the human and the radar sensor system took the Nakagami-*m* channel model. The analytical model for the false alarm and the detection probabilities of the system have been derived. The proposed theoretical models for the SNR and detection probability match with the simulation and measurement results. These theoretical models have the potential to be used as good references for the hardware development of the vital-sign detection radar sensor system.

## 1. Introduction

The non-contact monitoring of vital signals (heart beat and breathing rate) has attracted much attention recently. In this vital-sign monitoring system, the transceivers send a radio microwave signal toward the human position and collect the reflected signal from the person in order to obtain useful information such as vital signs and human location. There are many different approaches. The Doppler radar system has attracted a great deal of interest. The first Doppler radar system was developed for medical applications in the 1970s [1]. This radar system worked at a 10 GHz frequency, and it alarmed when apnoea occurred. This system did not give the exact respiration of the patient, but it did give an alarm when the patient stopped breathing for 30 s.

The application of microwave radar systems in health care systems has attracted a great deal of research attention [2,3,4,5,6,7,8]. In [3], the authors discussed the potential of using an ultra-wideband (UWB) technique for the radar sensor system in biological platform applications. Cianca et al. considered frequency modulation ultra-wideband (FM-UWB) in monitoring heart beats and breathing rates. In their work, the important features for medical applications, such as penetration capability, precision ranging, and electromagnetic radiation, were regarded carefully. The FM-UWB radar showed significant advantages in detecting vital signals [4]. Similarly, in 2011, Domenico Zito et al. [5] investigated UWB system-on-chip radar sensors in 90 nm CMOS technology. This system could detect the heart beat and respiration of adults and babies and allowed for the continuous observation of a baby’s breathing rate. This system could detect the breathing rate at a distance of 45 cm for the respiratory disorder diagnosis purpose and could sense a driver falling asleep. In [7], CMOS technology was also employed with the novel scheme design *bondwire-interconnection* between the antenna and the Doppler radar sensor system. This system could detect from a distance of up to 105 cm. A new patent of mm-wave radar sensors for detecting driver lethargy was investigated by Brankovic et al. in 2018 [8]. Their system could calculate the fatigue probability of a driver on the basis of the heart beat and respiratory rate. The probability was then compared with the threshold to give the driver an alarm signal. In [6], Stefano et al. reviewed a different type of micro-radar system for medical applications. In their research, the different types of radar sensor systems were examined carefully from continuous-wave (CW) to FM, UWB or hybrid radar systems.

Besides biomedical applications, a signal processing method for the vital-sign-detecting radar sensor system is critical [9,10,11]. In [9], Choi et al. showed that the multi-human detection algorithm could detect multiple people sequentially by detecting the first single cluster and repeating the process for the adjacent cluster. The signal-to-noise ratio (SNR) is one of the most important parameters of the radar system. The theoretical calculation of the SNR in the Doppler radar system should be considered and compared with the measured result to validate the theoretical considerations [10]. A denoising method for through-wall vital signs was proposed by Xiaolin et al. [11]. Several techniques were employed in this research, including short-time Fourier transform (STFT) and empirical mode decomposition (EEMD). This processing method can reduce clutter and is straightforward to implement.

Finding survivors buried under rubble is a crucial application of a Doppler radar sensor system [12,13]. In such an application, the microwave beam must penetrate deeply through the debris or rubble to find living victims. Therefore, *L* and *S* bands seem to be good choices. Kun-Mu Chen et al. [12] built systems and conducted experiments at two frequencies, 450 and 1150 MHz, and found that the 1150 MHz system operated better when the debris had metallic wires. In 2016, F. JalaliBidgoli et al. [13] proposed a similar system operating at a 1150 MHz frequency with the focus on minimizing clutter and noise [13]. This system is portable and can detect a victim below a depth of 1.5 m. Contrary to the earlier work, the transmission link between the radar system and the human body is a Friis channel model. This model implies a line-of-sight (LOS) connection between the radar sensor system and the subject. For the purpose of finding survivors, the system has to operate through different materials. Clearly, the LOS channel model is not adequate to describe the environment in which the radar system is likely to be used.

This paper presents research that considers the previous investigations and the drawbacks. In this research work, the SNR is carefully taken into account with respect to the Nakagami-*m* channel model and its special case (Rayleigh fading channel model). This model was chosen because it can more adequately describe realistic situations [14]. The Nakagami-*m* fading channel model can be used for a wide range of radio links, from non-fading channels to land-mobile or indoor-mobile prorogations. This channel can also serve as a LOS/non-line-of-sight (NLOS) channel model [15,16]. In comparison with previous studies, in terms of a channel radio link between the vital-sign-detecting radar system and the human, this research considers a more realistic and common channel model, the Nakagami-*m*, for the vital-sign-detecting radar sensor system. To the best of the authors’ knowledge, this is the first work to use the SNR at the base band under the Nakagami-*m* radio channel link between a living person and the radar sensor system. Moreover, the detection/false alarm probabilities of the vital-sign-detecting radar sensor system have been established, simulated and analyzed.

## 2. System Model

**Figure 1:**
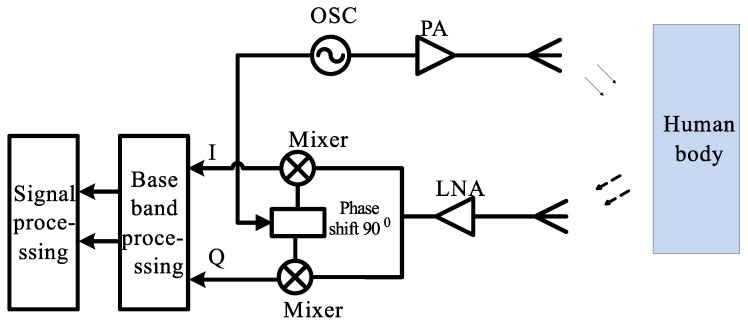
System diagram.

The working principle of the proposed vital-sign-detecting radar sensor system is presented using the block diagram shown in Figure 1. The transmitting antenna sends continuous waves to the human and waits for the reflected signals from the human body. At the human’s chest, the RF signal is modulated by the periodic displacement of the chest before being reflected back to the receiving antenna [17]. At the receiver, the signal is amplified by a low-noise amplifier (LNA) and is then sent to the phase detector. In this diagram, the in-phase (I) and quadrature (Q) phase detectors are used to reduce the null-point problem.

The transmitted signal from the vital-sign-detecting radar sensor system can be described by

(1) $$\begin{array}{c} Y_{T}=\sqrt{2P_{T}}\cos\left(2\pi ft+\phi \left(t\right)\right) \end{array}$$

where $P_{T}$ is the power of the transmitter, *f* is the carrier frequency, *t* is the transpiring time, and $\phi \left(t\right)$ is the phase noise. The initial phase of the transmitting signal is assumed to be zero. The signal $Y_{T}$ is then reflected by the human’s chest. The movement of the chest can be considered as the periodic signal $x\left(t\right)$. The distance from the human’s location to the antennae of the radar sensor system is *d* (m). The received signal $Y_{R}$ can be expressed as follows [18]:

(2) $$\begin{array}{c} Y_{R}=A_{R}\cos\left(2\pi ft-\frac{4\pi d}{\lambda }-\frac{4\pi x\left(t\right)}{\lambda }+\phi \left(t-\frac{2d}{c}\right)\right) \end{array}$$

where *c* is the light velocity, λ is the wavelength of the signal, and $A_{R}$ is the return signal amplitude that depends on te transmitted power, the gain of the antennae, the radar cross-section, and reflection loss from the human body and environment (channel links from the radar to the human body and from the human body to the radar).

In fact, in practice, the transmitting antenna is quite close to the receiving antenna, and thus the leakage noise is unavoidable. Moreover, the radar system also receives the reflected signal from the stationary part of the human and other clusters [17]. Therefore, the stable reflected signal should be considered at the receiving antenna. The receiving signal can be rewritten as

(3) $$\begin{array}{cc} Y_{R} & =A_{R}\cos\left(2\pi ft-\frac{4\pi d}{\lambda }-\frac{4\pi x\left(t\right)}{\lambda }+\phi \left(t-\frac{2d}{c}\right)\right)\\ & +A_{leak}\cos\left(2\pi f\left(t-t_{l}\right)+\phi \left(t-t_{l}\right)\right)\\ & +A_{c}\cos\left(2\pi ft-\frac{4\pi d_{0}}{\lambda }+\phi \left(t-\frac{2d}{c}\right)\right) \end{array}$$

where $A_{leak}$ is the amplitude of the leakage signal, $t_{l}$ is the time delay between the transmitting leakage signal and the local oscillator (LO) signal, and $A_{c}$ is the amplitude of the stationary cluster signal.

In previous studies, channel links between the radar sensor system and the human were considered as in free space [10]. This model implied that there were LOS links between the radar system and the human. However, the vital-sign-detecting radar system has to operate through the NLOS channel. Because of this reason, in this study, the Nakagami-*m* channel model was chosen as the channel model between the radar sensor system and the human. This channel model was considered to be close to the practical situation and could model both LOS and NLOS environments [14]. On the basis of the theory of radar systems [19], the amplitude of the receiving signal $A_{R}$ $\left[Volt\right]$ can be formulated as follows [20]:

(4) $$\begin{array}{c} A_{R}=\sqrt{\frac{2P_{T}G_{T}G_{R}\sigma_{a}\rho_{r}\rho_{t}r\lambda^{2}|g_{a}{|}^{2}{\left|g_{b}\right|}^{2}}{{\left(4\pi \right)}^{3}d^{2\alpha }}} \end{array}$$

where $G_{T}=10^{\frac{G_{T}\left[dBi\right]}{10}}$ and $G_{R}=10^{\frac{G_{R}\left[dBi\right]}{10}}$ are the gains of the transmitting antenna and receiving antenna, respectively; λ (m) is the wavelength of the signal; $\rho_{r}$ and $\rho_{t}$ are the radiated efficiencies of the receiving and transmitting antennae; *r* is the reflection coefficient of the human body; $\sigma_{a}$ (m^2^) is the radar cross-section (RCS) of the human’s chest; and α is the path loss. The channel coefficients for the links from the radar to the human body and from the human body to the radar are $g_{a}$ and $g_{b}$, respectively.

The leakage amplitude can be estimated as follows [21]:

(5) $$\begin{array}{c} A_{leak}=\sqrt{2\eta_{leak}P_{T}} \end{array}$$

and

(6) $$\begin{array}{c} A_{c}=\sqrt{\frac{2P_{T}G_{T}G_{R}\sigma_{c}\rho_{r}\rho_{t}r\lambda^{2}|g_{a}{|}^{2}{\left|g_{b}\right|}^{2}}{{\left(4\pi \right)}^{3}d^{2\alpha }}} \end{array}$$

where $G_{rx}$ is the gain of the receiver, and $\sigma_{c}$ is the RCS of other parts of the human body.

The receiving signal $Y_{R}$ is then mixed with the LO signal to obtain the intermediate frequency (IF) signal. As can be seen from Figure 1, I & Q demodulation is applied to reduce the null-point problem [22]. The output signals are then fed to the bandpass filter (BPF). The low- and high-end frequencies of the BPF are expressed as $f_{L}$ and $f_{H}$, respectively.

The base band can be written as follows [21]:

(7) $$\begin{array}{cc} B(t) & =A_{R}\sqrt{G_{RX}}\cos\left[\theta_{0}+\frac{4\pi x\left(t\right)}{\lambda }+▵\phi_{s}\left(t\right)\right]\\ & +A_{c}\sqrt{G_{RX}}\cos\left[\theta_{0}+▵\phi_{c}\left(t\right)\right]\\ & +A_{leak}\sqrt{G_{RX}}\cos\left[\theta_{l}+▵\phi_{l}\left(t\right)\right]+n\left(t\right)+n_{1/f}\left(t\right) \end{array}$$

where $\theta_{0}$ is a constant phase shift due to the distance from the human body to the radar system; $\theta_{l}$ is a constant phase shift that depends on the delay time $t_{l}$; $▵\phi_{s}\left(t\right)$, $▵\phi_{c}\left(t\right)$, and $▵\phi_{l}\left(t\right)$ are the residual phase noise of the reflected vital signals (respiratory signal and heart signal), clutter signal, and antennae leakage signal, respectively; n(t) and $n_{1/f}\left(\right)$ are the additive white Gaussian noise (AWGN) and base-band 1/f noise. The residual phase noise in Equation (7) can be calculated as follows [21]:

(8) $$\begin{array}{c} ▵\phi_{s}\left(t\right)\approx ▵\phi_{c}\left(t\right)=\phi \left(t\right)-\phi \left(t-\frac{2d}{c}\right) \end{array}$$

(9) $$\begin{array}{c} ▵\phi_{l}\left(t\right)=\phi \left(t\right)-\phi \left(t-▵t_{l}\right) \end{array}$$

In the following sections, the SNR is considered carefully, and the comparison between the theoretical calculation and the measurement is examined.

## 3. SNR Analysis

### 3.1. Signal Power

In the case in which the optimal demodulation is achieved, the receiving power at the output of the mixer can be given as follows [10,21,22]:

(10) $$\begin{array}{cc} S_{B} & =A_{R}^{2}G_{RX}{\left(\frac{4\pi x\left(t\right)}{\lambda }\right)}^{2}\\ & =\frac{2P_{T}G_{T}G_{R}\sigma_{a}\rho_{r}\rho_{t}r\lambda^{2}{\left|g_{a}\right|}^{2}{\left|g_{b}\right|}^{2}}{{\left(4\pi \right)}^{3}d^{2\alpha }}G_{RX}\frac{16\pi^{2}\bar{x^{2}\left(t\right)}}{\lambda^{2}}\\ & =\frac{P_{T}G_{T}G_{R}\sigma_{a}\rho_{r}\rho_{t}r{\left|g_{a}\right|}^{2}{\left|g_{b}\right|}^{2}G_{RX}\bar{x^{2}\left(t\right)}}{2\pi d^{2\alpha }} \end{array}$$

In this work, the main sources of noise have been considered carefully: residual phase noise, AWGN, and the base-band 1/f noise.

### 3.2. Residual Phase Noise

Clearly, phase noise degrades the performance of the Doppler radar system. The effect of this phenomenon is to spread energy of the clutter into the target frequency range. The spreading of clutter obscures the target signal and reduces the signal-to-clutter ratio [23]. In this section, the residual phase noise power is calculated. The residual phase noise consists of phase noise by the antennae leakage signal and the clutter. As defined in [10,21,23], the base-band noise spectral density ($S_{▵\phi }\left(f_{0}\right)$) is derived from the RF phase noise spectral density $S_{\theta }\left(f_{0}\right)$:

(11) $$\begin{array}{c} S_{▵\phi }\left(f_{0}\right)=S_{\phi }\left(f_{0}\right)4sin^{2}\left(2\pi \frac{df_{0}}{c}\right)\approx S_{\phi }\left(f_{0}\right)\left(\frac{16\pi^{2}d^{2}f_{0}^{2}}{c^{2}}\right) \end{array}$$

$S_{\theta }\left(f_{0}\right)$ can be derived from the phase noise at 1 Hz, $S_{\theta }\left(1\right)$, if the close-in phase noise has a −30 dB/decade slope, as follows [21]:

(12) $$\begin{array}{c} S_{\phi_{s}}\left(f_{0}\right)\approx \frac{S_{\phi }\left(1\right)}{{\left(1Hz\right)}^{-3}}f_{0}^{-3} \end{array}$$

On the basis of Equations (11) and (12), $S_{▵\phi_{c}}\left(f_{0}\right)$ can be given:

(13) $$\begin{array}{c} S_{▵\phi_{s}}\left(f_{0}\right)\approx \frac{S_{\phi }\left(1\right)}{{\left(1Hz\right)}^{-3}}f_{0}^{-3}\left(\frac{16\pi^{2}d^{2}f_{0}^{2}}{c^{2}}\right) \end{array}$$

The mean square of the residual phase noise in the time domain is calculated through the integral of the spectrum over the frequency range $\left[f_{L}tof_{H}\right]$ of the BPF [21]:

(14) $$\begin{array}{c} \bar{▵\phi_{s}{\left(t\right)}^{2}}=\int_{f_{L}}^{f_{H}}S_{▵\phi_{s}}\left(f_{0}\right)df_{0}=16\pi^{2}{\left(1Hz\right)}^{3}S_{\phi }\left(1\right)\frac{d^{2}}{c^{2}}ln\left[\frac{f_{H}}{f_{L}}\right] \end{array}$$

Finally, the residual phase noise power ($N_{▵\phi_{s}}$) of the received signal can be calculated [21]:

(15) $$\begin{array}{cc} N_{▵\phi_{s}} & \approx A_{R}^{2}G_{RX}\bar{▵\phi_{s}{\left(t\right)}^{2}}\\ & \approx \frac{2P_{T}G_{T}G_{R}\sigma_{a}\rho_{r}\rho_{t}r\lambda^{2}{\left|g_{a}\right|}^{2}{\left|g_{b}\right|}^{2}}{{\left(4\pi \right)}^{3}d^{2\alpha }}G_{RX}16\pi^{2}{\left(1Hz\right)}^{3}S_{\phi }\left(1\right)\frac{d^{2}}{c^{2}}ln\left[\frac{f_{H}}{f_{L}}\right]\\ & \approx \frac{P_{T}G_{T}G_{R}\sigma_{a}\rho_{r}\rho_{t}r\lambda^{2}{\left|g_{a}\right|}^{2}{\left|g_{b}\right|}^{2}}{2\pi d^{2\left(\alpha -1\right)}f^{2}}G_{RX}S_{\phi }\left(1\right)ln\left[\frac{f_{H}}{f_{L}}\right] \end{array}$$

By following similar steps to Equations (11)–(15), the residual phase noise power ($N_{▵\phi_{c}}$) of the clutter signal can be calculated:

(16) $$\begin{array}{c} N_{▵\phi_{c}}\approx \frac{P_{T}G_{T}G_{R}\sigma_{c}\rho_{r}\rho_{t}r\lambda^{2}|g_{a}{|}^{2}{\left|g_{b}\right|}^{2}}{2\pi d^{2\left(\alpha -1\right)}f^{2}}G_{RX}S_{\phi }\left(1\right)ln\left[\frac{f_{H}}{f_{L}}\right] \end{array}$$

This then turns to antennae leakage phase noise; the base-band spectral density of antennae leakage phase noise $S_{▵\phi_{l}}\left(f_{0}\right)$ is given as

(17) $$\begin{array}{c} S_{▵\phi_{l}}\left(f_{0}\right)\approx \frac{S_{\phi }\left(1\right)}{{\left(1Hz\right)}^{-3}}f_{0}^{-3}\left(\frac{16\pi^{2}▵t_{l}^{2}f_{0}^{2}}{4}\right) \end{array}$$

The power of the antennae leakage phase noise ($N_{▵\phi_{l}}$) can be expressed as

(18) $$\begin{array}{cc} N_{▵\phi_{l}} & \approx A_{leak}^{2}G_{RX}\bar{▵\phi_{l}{\left(t\right)}^{2}}\\ & \approx A_{leak}^{2}G_{RX}\int_{f_{L}}^{f_{H}}S_{▵\phi_{l}}\left(f_{0}\right)df_{0}\\ & \approx A_{leak}^{2}G_{RX}16\pi^{2}{\left(1Hz\right)}^{3}S_{\phi }\left(1\right)\frac{▵t_{l}^{2}}{2^{2}}ln\left[\frac{f_{H}}{f_{L}}\right]\\ & \approx 32\eta_{leak}P_{T}G_{RX}\pi^{2}S_{\phi }\left(1\right)\frac{▵t_{l}^{2}}{2^{2}}ln\left[\frac{f_{H}}{f_{L}}\right] \end{array}$$

### 3.3. Additive White Gaussian Noise

AWGN is the main noise at the receiver’s input, and afterwards, this noise is converted to the baseband with the power ($N_{W}$) as follows [10,21]:

(19) $$\begin{array}{c} N_{W}=8G_{RX}\left(kTB\right)\left(NF\right) \end{array}$$

where $G_{RX}=10^{\frac{G_{RX}\left[dBi\right]}{10}}$ is the gain of the receiver, *B* (Hz) is the bandwidth, *T* (K) is the absolute temperature, *k* is Boltzman’s constant, and $NF=10^{\frac{NF\left[dB\right]}{10}}$ is the noise figurer of the receiver.

### 3.4. 1/f Noise

The mixer is chosen to minimize the 1/f noise. The power of the 1/f noise ($N_{1/f}$) can be designated by the noise power ($P_{1/f}\left(1\right)$) in a 1 Hz bandwidth centered at 1 Hz:

(20) $$\begin{array}{c} N_{1/f}=\int_{f_{L}}^{f_{H}}P_{1/f}\left(1\right)f^{-1}df=P_{1/f}\left(1\right)ln\left(\frac{f_{H}}{f_{L}}\right) \end{array}$$

### 3.5. SNR

The noise sources are uncorrelated, and the powers of the noise are added together. The SNR is written as

(21) $$\begin{array}{c} SNR=\frac{S_{B}}{N_{▵\phi_{s}}+N_{▵\phi_{c}}+N_{▵\phi_{l}}+N_{W}+N_{1/f}} \end{array}$$

## 4. Detection and False Alarm Probabilities

In this section, the analysis and simulation results of the detection probability ($P_{D}$) and false alarm probability ($P_{f}$) of the proposed system are presented. Estimations of the detection/false alarm probabilities are very important for the vital-sign-detecting radar system. The decision hypothesis is detected when the receiving signal and noise are greater than the threshold value $P_{th}$. The decision is a false alarm when the power of the noise is greater than the threshold $P_{th}$ [9,19]. The false alarm/detection probabilities are estimated under two hypotheses, $H_{0}$ and $H_{1}$. $H_{0}$ corresponds to the case that the vital signal is not presented, and $H_{1}$ is the opposite case.

### 4.1. Detection Probability

The detection probability can be defined as follows:

(22) $$\begin{array}{c} P_{D}=P_{r}\left\{S_{B}+N_{▵\phi_{s}}+N_{▵\phi_{c}}+N_{▵\phi_{l}}+N_{W}+N_{1/f}\geq P_{th}|H_{1}\right\} \end{array}$$

By using $C=\frac{P_{T}G_{T}G_{R}\sigma_{a}\rho_{r}\rho_{t}rG_{RX}\bar{x^{2}\left(t\right)}}{2\pi d^{2\alpha }}$ and $D=\frac{P_{T}G_{T}G_{R}\rho_{r}\rho_{t}r\lambda^{2}}{2\pi d^{2\left(\alpha -1\right)}f^{2}}G_{RX}S_{\phi }\left(1\right)$, $P_{D}$ can be rewritten as follows:

(23) $$\begin{array}{cc} P_{D} & =P_{r}\left\{C|g_{a}{|}^{2}|g_{b}{|}^{2}+D\sigma_{a}|g_{a}{|}^{2}|g_{b}{|}^{2}+D\sigma_{c}|g_{a}{|}^{2}{\left|g_{b}\right|}^{2}+N_{▵\phi_{l}}+N_{W}+N_{1/f}\geq P_{th}\right\}\\ & =P_{r}\left\{\left(C+D\sigma_{a}+D\sigma_{c}\right)|g_{a}{|}^{2}{\left|g_{b}\right|}^{2}+N_{▵\phi_{l}}+N_{W}+N_{1/f}\geq P_{th}\right\}\\ & =1-P_{r}\left\{|g_{a}{|}^{2}{\left|g_{b}\right|}^{2}\leq \frac{P_{th}-\left(N_{▵\phi_{l}}+N_{W}+N_{1/f}\right)}{C+D\sigma_{a}+D\sigma_{c}}\right\} \end{array}$$

The channel model between the radar system and the human body is Nakagami-*m*, where *m* is the fading severity parameter. We assume that $|g_{a}^{2}|$ and $|g_{b}^{2}|$ are independent gamma random variables with the severity fading factor $m_{a}$ and $m_{b}$; in the case m=1, the Nakagami-*m* model becomes the Rayleigh fading channel model. By applying the work from [20], the detection probability can be given as

(24) $$\begin{array}{c} P_{D}=2\sqrt{\frac{P_{th}-\left(N_{▵\phi_{l}}+N_{W}+N_{1/f}\right)}{\left(C+D\sigma_{a}+D\sigma_{c}\right)\mu_{a}\mu_{b}}}K1\left(2\sqrt{\frac{P_{th}-\left(N_{▵\phi_{l}}+N_{W}+N_{1/f}\right)}{\left(C+D\sigma_{a}+D\sigma_{c}\right)\mu_{a}\mu_{b}}}\right) \end{array}$$

where $\mu_{a}$ and $\mu_{b}$ are the mean values of $|g_{a}^{2}|$ and $|g_{b}^{2}|$, respectively. K1(.) is the first-order modified Bessel function of the second kind, $K1\left(x\right)=\int_{0}^{\infty }e^{-xcosh\left(t\right)}cosh\left(t\right)dt$ [24].

When it comes to the normal case of the Nakagami-*m* channel model, the detection probability of the sensor system can be calculated on the basis of the distribution of the products of two independent variables $|g_{a}^{2}|$ and $|g_{b}^{2}|$. Associated with the probability theory in [25], $P_{D}$ can be calculated as

(25) $$\begin{array}{c} P_{D}=1-u2^{1-m_{a}-m_{b}}Dm_{1}+m_{2}-1,m_{1}-m_{2}\left(2\sqrt{\frac{P_{th}-\left(N_{▵\phi_{l}}+N_{W}+N_{1/f}\right)}{C+D\sigma_{a}+D\sigma_{c}}}\right) \end{array}$$

where $u=2\tau {\left(m_{1}\right)}^{-1}\tau {\left(m_{2}\right)}^{-1}$ and $D_{\mu ,v}\left(x\right)=\int_{0}^{x}x^{\mu }K_{v}\left(x\right)dx$; $\tau \left(.\right)$ and $K\left(.\right)$ are the gamma function and Bessel function, respectively. These functions can be found in [26] and calculated by available software such as Matlab or Wolfram. In the special case $m_{a}$ = $m_{b}$ = I + 1 (where I = 0, 1, 2, ..... ), $P_{D}$ is given as follows [25]:

(26) $$\begin{array}{c} P_{D}=2^{-2i}{\left(i!\right)}^{-1}\sum_{j=0}^{i}{\left(2\sqrt{\frac{P_{th}-\left(N_{▵\phi_{l}}+N_{W}+N_{1/f}\right)}{C+D\sigma_{a}+D\sigma_{c}}}\right)}^{2i+1-j}\times \\ K_{j+1}\left(2\sqrt{\frac{P_{th}-\left(N_{▵\phi_{l}}+N_{W}+N_{1/f}\right)}{C+D\sigma_{a}+D\sigma_{c}}}\right)2^{j}/\left(i-j\right)! \end{array}$$

### 4.2. False Alarm Probability

Similarly, the false alarm probability is given as

(27) $$\begin{array}{cc} P_{f} & =P_{r}\left\{N_{▵\phi_{s}}+N_{▵\phi_{c}}+N_{▵\phi_{l}}+N_{W}+N_{1/f}\geq P_{th}|H_{0}\right\}\\ & =1-P_{r}\left\{|g_{a}{|}^{2}{\left|g_{b}\right|}^{2}\leq \frac{P_{th}-\left(N_{▵\phi_{l}}+N_{W}+N_{1/f}\right)}{D\sigma_{a}+D\sigma_{c}}\right\} \end{array}$$

Under the Rayleigh fading channel model, the false alarm probability can be expressed as

(28) $$\begin{array}{c} P_{f}=2\sqrt{\frac{P_{th}-\left(N_{▵\phi_{l}}+N_{W}+N_{1/f}\right)}{\left(D\sigma_{a}+D\sigma_{c}\right)\mu_{a}\mu_{b}}}K1\left(2\sqrt{\frac{P_{th}-\left(N_{▵\phi_{l}}+N_{W}+N_{1/f}\right)}{\left(D\sigma_{a}+D\sigma_{c}\right)\mu_{a}\mu_{b}}}\right) \end{array}$$

Under the Nakagami-*m* channel model (m≥1/2), the false alarm probability can be given as

(29) $$\begin{array}{c} P_{f}=1-u2^{1-m_{a}-m_{b}}Dm_{1}+m_{2}-1,m_{1}-m_{2}\left(2\sqrt{\frac{P_{th}-\left(N_{▵\phi_{l}}+N_{W}+N_{1/f}\right)}{D\sigma_{a}+D\sigma_{c}}}\right) \end{array}$$

In the case $m_{a}$ = $m_{b}$ = I + 1 (where I = 0, 1, 2, ..... ), $P_{f}$ can be calculated as follows [25]:

(30) $$\begin{array}{c} P_{f}=2^{-2i}{\left(i!\right)}^{-1}\sum_{j=0}^{i}{\left(2\sqrt{\frac{P_{th}-\left(N_{▵\phi_{l}}+N_{W}+N_{1/f}\right)}{D\sigma_{a}+D\sigma_{c}}}\right)}^{2i+1-j}\\ \times K_{j+1}\left(2\sqrt{\frac{P_{th}-\left(N_{▵\phi_{l}}+N_{W}+N_{1/f}\right)}{D\sigma_{a}+D\sigma_{c}}}\right)2^{j}/\left(i-j\right)! \end{array}$$

## 5. Simulation Result

In this section, the SNR and detection/false alarm probabilities are discussed in terms of simulation and analysis. The values used for simulation and analysis are given in Table 1.

### 5.1. SNR and Detection/False Alarm Probabilities under Rayleigh Fading Channel

**Figure 2:**
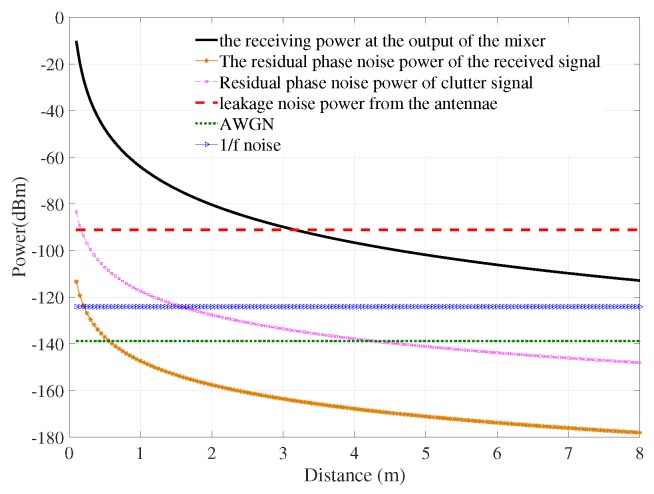
Power of signal and noise of the system.

Firstly, a special case of the Nakagami-1 (Rayleigh fading channel) channel model has been considered. This model implied that there was no LOS transmission between the human and the radar system. The power of the receiving signal and different types of noise are shown in Figure 2. The leakage noise was relatively high, around −90 dBm, while the AWGN and 1/f noise were lower than 120 dBm. The residual phase noise power of the received signal had less effect on the system than the residual phase noise power of the clutter signal; both decreased with the distance increase. It is noted from this simulation that with the transmitting power of 0 dBm, the receiving power was greater than the power of each type of noise when the distance was less than 3 m. This figure gives an important idea to enhance the system. The most dominant noise was leakage noise; this type of noise is defined by the isolation level between the transmitting and receiving antennae. Therefore, this noise could be minimized by hardware development and signal processing techniques.

**Figure 3:**
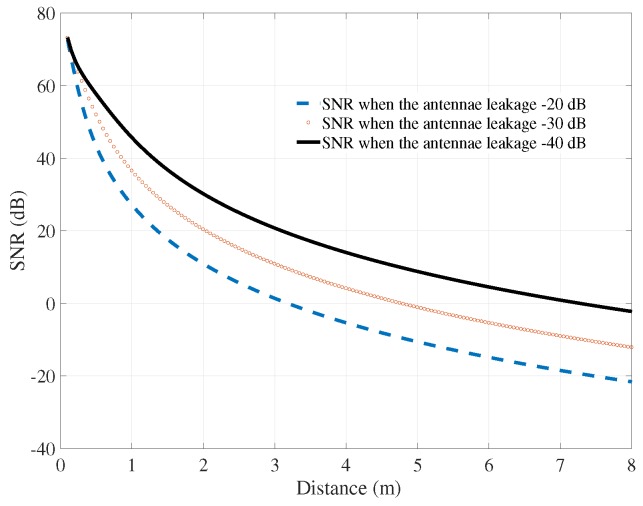
Signal-to-noise ratio (SNR) versus the distance from the radar system to the human body.

Besides the power of the signal and noise, the SNR is the most important feature of the radar sensor system. The overall SNR of system is displayed in Figure 3. The first point to note is that the SNR decreased whenever the distance between the human body to the radar system and the antennae leakage increased. At the level of antennae leakage of −20 dB, the SNR reduced from around 30 dB at a distance of 1 m to −20 dB at a distance of 7 m. When the leakage between the two antennae was at −40 dB, the SNR at the same distance increased to 0 dB.

**Figure 4:**
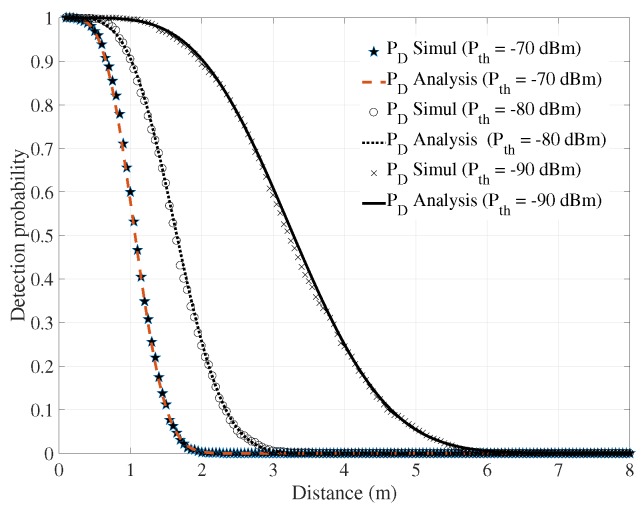
Detection probabilities of the system under different threshold levels of receiver filter.

Figure 4 displays the detection probability of the system at three different levels of the threshold power of the receiver’s filter, −70, −80, and −90 dBm. The lowest threshold value gave the highest detection probability. When the distance increased, the detection probability decreased. The threshold power of the receiver’s filter defined the steepness of the detection probability line.

**Figure 5:**
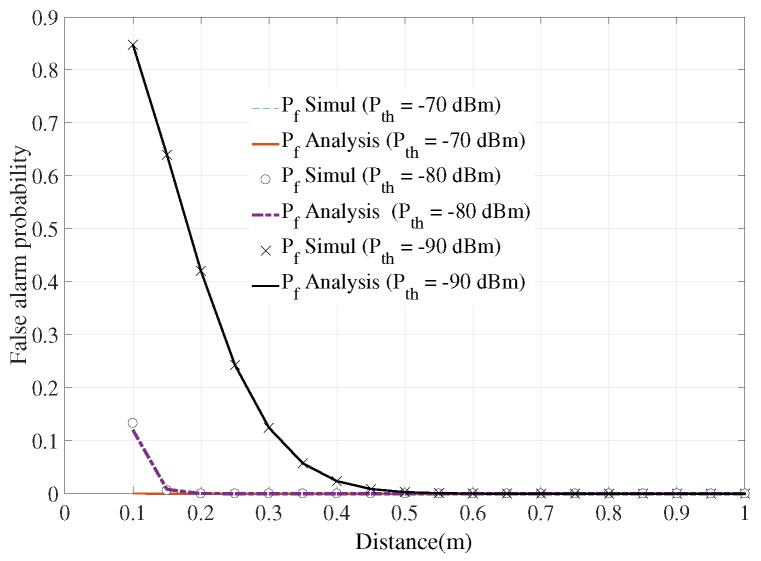
False alarm of the system under different threshold levels of receiver filter.

**Figure 6:**
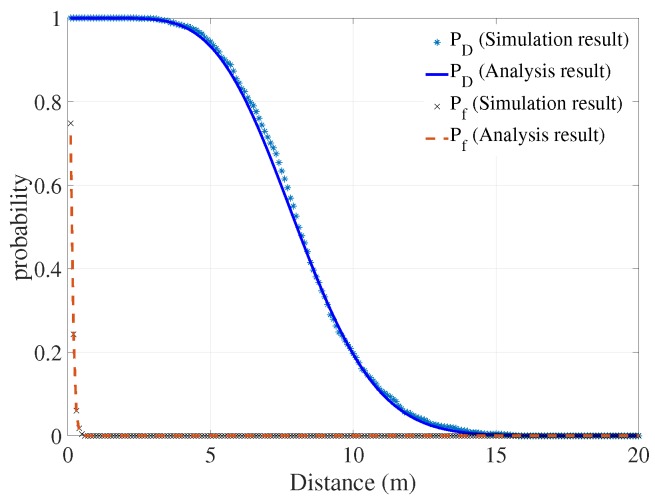
False alarm/detection probabilities of the system under Nakagami-2 channel model.

To evaluate the accuracy of the vital-sign-detecting radar sensor, the false alarm should be taken into account. Figure 5 shows that when the threshold power of the receiver’s filter was set at −70 dBm, the false alarm probability was high at distances of less than 50 cm. This phenomenon was due to the high level of phase noise when the radar was too close to the human body. When this threshold value reduced to −90 dBm, the false alarm moved to the zero level. The simulation and analysis results are presented in Figure 2, Figure 3, Figure 4 and Figure 5, Figure 6. The performance of the system is discussed under the Rayleigh Fading channel model. This model implied a NLOS link between the human body and the radar system.

### 5.2. Detection/False Alarm Probabilities of the System under the Nakagami-2 Channel Model

When the quality of the channel increased, for example, when the fading factor m= 2, in comparison with the Rayleigh fading link, the detection probability increased significantly. At a distance of around 7 m, the detection probability was approximately 80% as shown in Figure 6. The false alarm detection probability was relatively high at a close distance (less than 50 cm) because of the high level of phase noise.

## 6. Measurement Results

**Figure 7:**
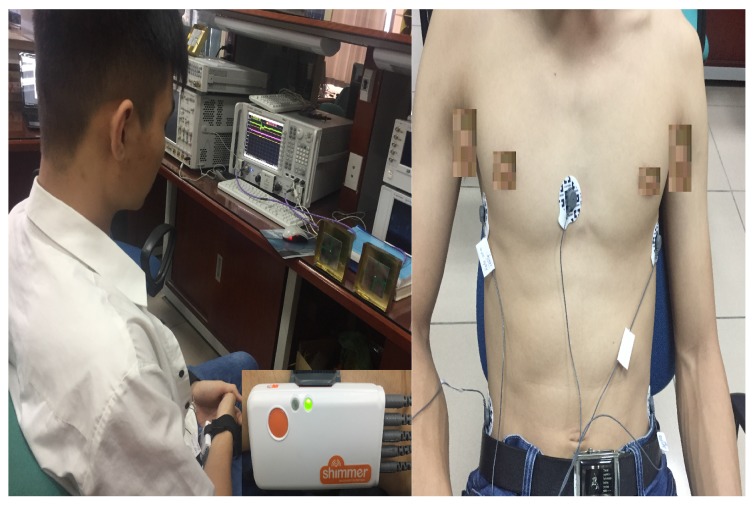
Measurement setup to detect the breathing rate.

**Figure 8:**
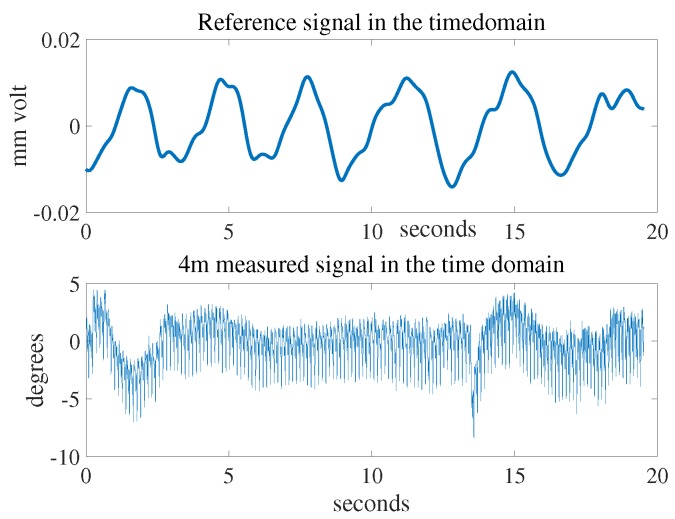
Receiving signals in time domain.

**Figure 9:**
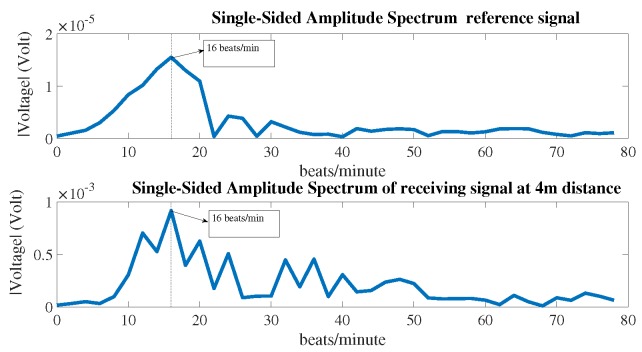
Receiving signals in frequency domain.

**Figure 10:**
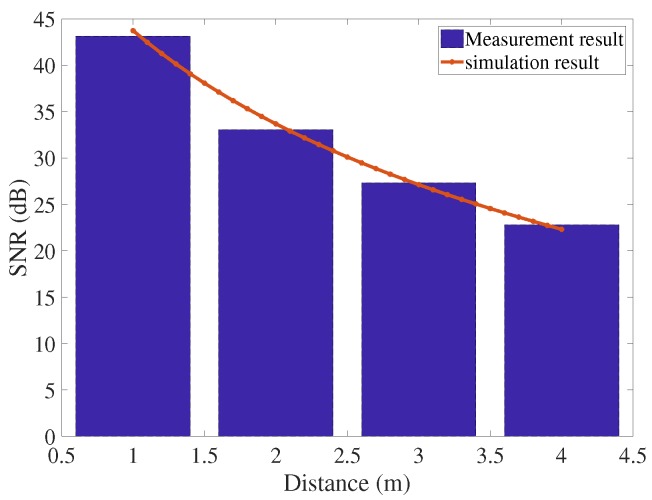
Signal-to-noise ratio (SNR) of the system.

**Figure 11:**
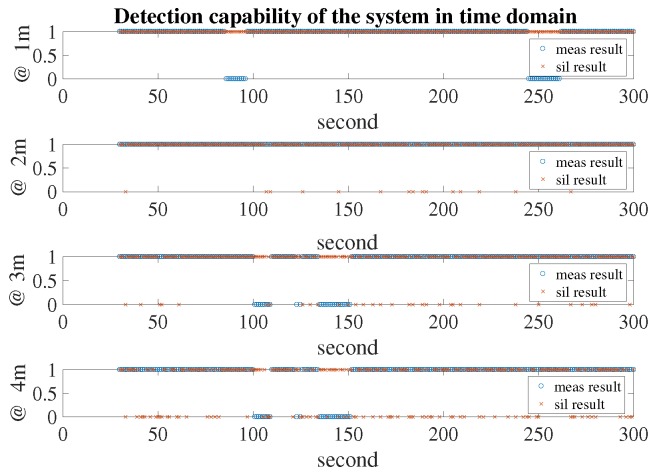
Detection capability of the system in time domain.

**Figure 12:**
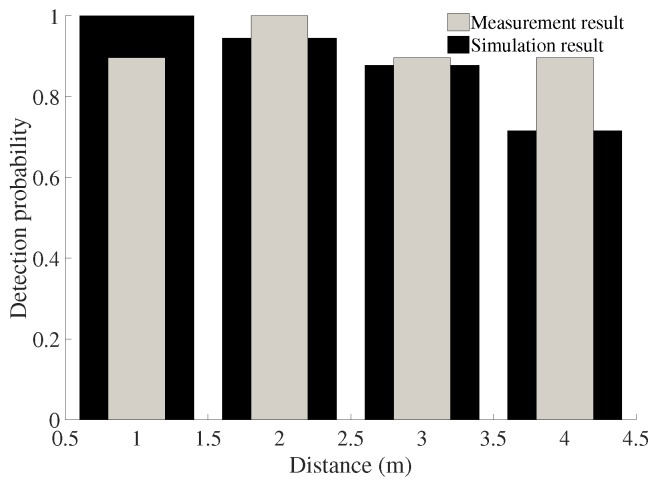
Detection probability of the system at different distances.

This section presents the measurement results of detecting the respiratory rate of humans. The SNR and the detection capability/probability were considered and compared to those of the theoretical models. The measurement was a setup based on the N5244A PNA−X Microwave Network Analyzer, 43.5 GHz. Two ports of this device were connected to two antennae, as shown in Figure 7. Five people aged from 21 to 25 participated in this experiment. The objects (humans) were required to sit in front of the antennae; one antenna played the role of the transmitting antenna, and the other was the receiving antenna. The operating frequency of the system was set up in *L* band to obtain higher penetration through different environments [27]. In this study, the frequency was chosen as the optimal operating frequency (1.6 GHz) of the microstrip/path antenna. The antennae’s size was 10 cm × 10 cm with a gain of 5 dBi. For each distance (1, 2, 3, and 4 m), objects were measured over 5 min continuously in the normal electronic lab. The internal phase detector of the N5244A PNA−X Microwave Network Analyzer was utilized to find the phase shift of the receiving signal. This signal was then processed in Matlab to find the breathing rate. At the same time, the object’s breathing rate was measured by the wearable sensor Shimmer 3 ECG/EMG with five probe leads connected, as shown in Figure 7; the output signals of the Shimmer 3 were sent to the computer by a Bluetooth connection. The measurement results are shown in Table 2. The breathing rates at different distances were close to the reference values. For the rescue detection purpose, the deviation around the beats per minute was acceptable. Regarding the SNR values, at every distance, the measured results were close to the simulation results. The difference was only around 1.5 dBm. The measurement result of the SNR of the second object was the closest to the simulation outcome and the mean values of the five cases. The detailed measured results of object 2 are presented in Figure 8, Figure 9, Figure 10, Figure 11 and Figure 12.

Figure 8 shows the reference signal and the measured signal at a distance of 4 m in the time domain by the radar system. These signals were then processed by the fast Fourier transform (FFT) to find the breathing rate. The signals in the frequency domain can be seen in Figure 9. The breathing rate of the measured person was 16 beats per minute. The window size of the FFT was 30 s and moved forward every second; the number of samples in each second was 256. The SNR and the detection capability of the system were evaluated at each second after the first 30 s.

The SNR of the breathing signal was calculated by the mean value of the signal in the following range: (center frequency − 4 beats) to (center frequency + 4 beats), over the mean value of the remaining signal from 5 to 40 beats/min [28]. The breathing rate of the person was measured at four positions (1, 2, 3, and 4 m). The SNR of the system at each distance was calculated by the mean values of the SNR in each window. Figure 10 shows that the measured result was in line with the theoretical analysis in the case of the Nakagami-2 model. In this situation, the fading parameter was 2, corresponding the LOS connection between the radar system and the human body. Figure 11 presents the detection capability of the system in the time domain.

In Figure 11, the detection capabilities of the system at four distances are presented. When the detection capability equalled 1, the radar system could detect the breathing rate of a human. As can be seen in Figure 11, at most time points, the measured result matched with the simulation outcome. However, when the object was closer to the radar system, the detection capability was higher and the false detection in the measured result was distributed over a period of time. For example, at a distance of 3 m, the false detections occurred at around the 100th and 150th seconds, while the simulation showed that the false detection occurred randomly at different time points. This phenomenon could be explained by, for some duration in the laboratory environment, the noise level increasing significantly and negatively affecting the radar system; the simulation result, however, followed the statistical distribution—the errors occurred randomly at different time points.

The detection probability of the system is shown in Figure 12. At distances of 2 and 3 m, the measurement results were close to those of the theoretical model, while at a distance of 1 m, the detection probability was higher than that of the simulation result; the opposite result at a distance of 4 m was found. These differences may have appeared because the measuring time was not long enough. However, the theoretical detection probability model is a good reference to evaluate the performance of the radar sensor system in the long term.

## 7. Conclusions

In this paper, the SNR and the detection probability of the vital-sign-detecting radar sensor system have been considered and simulated. The measured result of the SNR was close to the theoretical calculation. This theoretical model is a basis for hardware development. The designer could optimize their design by choosing different parameters, such as leakage noise, phase noise, and so on, to obtain a high value of the SNR. In addition, on the basis of different types of noise, the detection probability has also been investigated in terms of simulation and analysis. The simulation followed the analytical models. When the system operated at close distances, the phase noise was relatively high and caused a false alarm problem. The threshold power of the receiver’s filter affected the alarm/detection probabilities. This value should be enhanced to improve the detection probability and reduce the false alarm probability. Moreover, the isolation between the transmitting and receiving antennae is an important factor to boost the SNR and detection probability of the system.

## Figures and Tables

**Table 1:** System parameters used for simulation.

Symbol	Description	Value
$P_{T}$	Power of transmitter	0 dBm
$G_{T}$	Transmitting antenna gain	5 dBi
$G_{R}$	Receiving antenna gain	5 dBi
$G_{RX}$	Gain of the receiver	10 dB
$\rho_{r}$	Radiated efficiency of receiving antenna	0.8
$\rho_{t}$	Radiated efficiency of transmitting antenna	0.8
*r*	Reflection coefficient of the human body	0.5
$\sigma_{a}$	RCS of the human’s chest	500 ${\mathrm{mm}}^{2}$
NF	Received noise figure	6 dB
$P_{1/f}\left(1\right)$	1/f power noise power at 1 Hz	−130 dBm/Hz
*T*	Absolute noise temperature	300
$f_{H}$	High frequency	5 Hz
$f_{L}$	Low frequency	0.1 Hz
$S_{▵}\left(1\right)$	Phase noise at 1 Hz intercept	60 dB/Hz
$\eta_{leak}$	Leakage between transmitting and receiving antennae	−20 dB
α	Path loss	2

**Table 2:** Respiratory rates and signal-to-noise ratio (SNR) from five objects at different distances.

Object	Breathing Rate					SNR							
	Ref	1 m	2 m	3 m	4 m	1 m		2 m		3 m		4 m	
						Sim.	Meas.	Sim.	Meas.	Sim.	Meas.	Sim.	Meas.
1	15	16	17	18	18	43.73	43.37	33.67	21.76	27.12	13.5	22.31	15.77
2	16	17	15	18	16	**43.73**	**43.13**	**33.67**	**33.03**	**27.12**	**27.32**	**22.31**	**21.77**
3	16	18	18	19	17	43.73	40.11	33.67	40.44	27.12	30.65	22.31	27.08
4	17	18	16	17	13	43.73	66.25	33.67	43.19	27.12	24.55	22.31	19.55
5	16	18	16	17	16	43.73	34.09	33.67	44.56	27.12	27.12	22.31	18.65
Mean values	16	17.4	16.4	17.8	16	**43.73**	**45.39**	**33.67**	**36.59**	**27.12**	**24.62**	**22.31**	**20.56**

## References

[1] Caro C., Bloice J. (1971). Contactless apnoea detector based on radar. Lancet.

[2] McEwan T.E. (1996). Body Monitoring and Imaging Apparatus and Method. US Patent.

[3] Bilich C.G. Bio-medical sensing using ultra wideband communications and radar technology: A feasibility study. Proceedings of the Pervasive Health Conference and Workshops.

[4] Cianca E., Gupta B. (2009). FM-UWB for communications and radar in medical applications. Wirel. Pers. Commun..

[5] Zito D., Pepe D., Mincica M., Zito F., Tognetti A., Lanatà A., De Rossi D. (2011). SoC CMOS UWB pulse radar sensor for contactless respiratory rate monitoring. IEEE Trans. Biomed. Circuits Syst..

[6] Pisa S., Pittella E., Piuzzi E. (2016). A survey of radar systems for medical applications. IEEE Aerosp. Electron. Syst. Mag..

[7] Chan C.H., Chou C.C., Chuang H.R. (2018). Integrated Packaging Design of Low-Cost Bondwire Interconnection for 60-GHz CMOS Vital-Signs Radar Sensor Chip With Millimeter-Wave Planar Antenna. IEEE Trans. Compon. Packag. Manuf. Technol..

[8] Branković V., Jovanović P., Mihajlović V., Savić M., Tasovac D. (2018). MM-Wave Radar Driver Fatigue Sensor Apparatus. US Patent.

[9] Choi J.W., Nam S.S., Cho S.H. (2016). Multi-Human Detection Algorithm based on an Impulse Radio Ultra-Wideband Radar System. IEEE Access.

[10] Droitcour A.D., Boric-Lubecke O., Kovacs G.T. (2009). Signal-to-noise ratio in Doppler radar system for heart and respiratory rate measurements. IEEE Trans. Microw. Theory Tech..

[11] Liang X., Zhang H., Ye S., Fang G., Gulliver T.A. (2018). Improved denoising method for through-wall vital sign detection using UWB impulse radar. Digit. Signal Process..

[12] Chen K.M., Huang Y., Zhang J., Norman A. (2000). Microwave life-detection systems for searching human subjects under earthquake rubble or behind barrier. IEEE Trans. Biomed. Eng..

[13] JalaliBidgoli F., Moghadami S., Ardalan S. (2016). A Compact Portable Microwave Life-Detection Device for Finding Survivors. IEEE Embed. Syst. Lett..

[14] Mishra M.K., Sood N., Sharma A.K. (2011). Efficient BER Analysis of OFDM System over Nakagami-m Fading Channel. Int. J. Adv. Sci. Technol..

[15] Alouini M.S., Simon M.K. (1999). Performance of coherent receivers with hybrid SC/MRC over Nakagami-m fading channels. IEEE Trans. Veh. Technol..

[16] Suraweera H.A., Smith P.J., Armstrong J. (2006). Outage probability of cooperative relay networks in Nakagami-m fading channels. IEEE Commun. Lett..

[17] Kuo H.C., Lin C.C., Yu C.H., Lo P.H., Lyu J.Y., Chou C.C., Chuang H.R. (2016). A Fully Integrated 60-GHz CMOS Direct-Conversion Doppler Radar RF Sensor with Clutter Canceller for Single-Antenna Noncontact Human Vital-Signs Detection. IEEE Trans. Microw. Theory Tech..

[18] Girbau D., Lazaro A., Ramos A., Villarino R. (2012). Remote sensing of vital signs using a doppler radar and diversity to overcome null detection. IEEE Sens. J..

[19] Mahafza B.R. (2013). Radar Systems Analysis and Design Using MATLAB Third Edition.

[20] Van N.T.P., Hasan S.F., Gui X., Mukhopadhyay S., Tran H. (2017). Three-Step Two-Way Decode and Forward Relay With Energy Harvesting. IEEE Commun. Lett..

[21] Jang B.J., Wi S.H., Yook J.G., Lee M.Q., Lee K.J. (2008). Wireless bio-radar sensor for heartbeat and respiration detection. Prog. Electromagn. Res. C.

[22] Droitcour A.D., Boric-Lubecke O., Lubecke V.M., Lin J., Kovacs G.T. (2004). Range correlation and I/Q performance benefits in single-chip silicon Doppler radars for noncontact cardiopulmonary monitoring. IEEE Trans. Microw. Theory Tech..

[23] Budge M., Burt M. Range correlation effects on phase and amplitude noise. Proceedings of the Southeastcon’93.

[24] Kreh M. (2012). Bessel Functions: Project for the Penn State—Gttingen Summer School on Number Theory. http://www.math.psu.edu/papikian/Kreh.pdf.

[25] Withers C.S., Nadarajah S. (2013). On the product of gamma random variables. Qual. Quant..

[26] Jeffrey A., Zwillinger D. (2007). Table of Integrals, Series, and Products.

[27] Grandjean G., Baghdadi N., Paillou P., Dreuillet P., Dubois P., Souyris J., Achache J. (2000). Radar penetration in soils: Towards a new system for subsurface Earth observation. Proceedings of a CEOS SAR Workshop, Toulouse, France, 26–29 October 1999.

[28] Tariq A. (2013). Vital Signs Monitoring Using Doppler Radar and on-Body Antennas. Ph.D. Thesis.

